# Osteocytic alterations in CKD-associated osteomalacia appear to differ according to aluminum deposition, suggesting distinct microenvironmental and canalicular phenotypes

**DOI:** 10.1093/jbmrpl/ziag167

**Published:** 2026-10-09

**Authors:** Julia B Vaz, Cleonice Silva, Ivone B Oliveira, Wagner Vasques Dominguez, Rosa Maria Affonso Moysés, Luciene M dos Reis, Vanda Jorgetti

**Affiliations:** LIM 16 – Laboratorio de Fisiopatologia Renal, Hospital das Clinicas HCFMUSP, Faculdade de Medicina, Universidade de Sao Paulo, Sao Paulo, SP 01246-903, Brasil; LIM 16 – Laboratorio de Fisiopatologia Renal, Hospital das Clinicas HCFMUSP, Faculdade de Medicina, Universidade de Sao Paulo, Sao Paulo, SP 01246-903, Brasil; LIM 16 – Laboratorio de Fisiopatologia Renal, Hospital das Clinicas HCFMUSP, Faculdade de Medicina, Universidade de Sao Paulo, Sao Paulo, SP 01246-903, Brasil; LIM 16 – Laboratorio de Fisiopatologia Renal, Hospital das Clinicas HCFMUSP, Faculdade de Medicina, Universidade de Sao Paulo, Sao Paulo, SP 01246-903, Brasil; LIM 16 – Laboratorio de Fisiopatologia Renal, Hospital das Clinicas HCFMUSP, Faculdade de Medicina, Universidade de Sao Paulo, Sao Paulo, SP 01246-903, Brasil; LIM 16 – Laboratorio de Fisiopatologia Renal, Hospital das Clinicas HCFMUSP, Faculdade de Medicina, Universidade de Sao Paulo, Sao Paulo, SP 01246-903, Brasil; LIM 16 – Laboratorio de Fisiopatologia Renal, Hospital das Clinicas HCFMUSP, Faculdade de Medicina, Universidade de Sao Paulo, Sao Paulo, SP 01246-903, Brasil

**Keywords:** osteomalacia, aluminum toxicity, osteocytes, CKD, mineral and bone disorder, bone biopsy

## Abstract

Osteomalacia is a severe mineralization defect characterized by increased osteoid matrix and delayed mineral apposition. In CKD, particularly in patients on hemodialysis (HD), aluminum deposition has been implicated as a risk factor. However, it remains unclear whether osteomalacia and aluminum-associated osteomalacia represent distinct variants. We compared clinical, biochemical, histomorphometric, and osteocytic features between HD patients with osteomalacia with and without aluminum deposition. This is a cross-section study of 27 HD patients with osteomalacia, 15 without and 12 with aluminum deposition, defined by solochrome azurine staining as stainable aluminum in ≥30% of the mineralization front. Clinical/biochemical data and bone histomorphometry were assessed. Osteocytic proteins, osteopontin (OPN), and apoptosis were evaluated by immunohistochemistry, globally and separately in mineralized matrix and osteoid. Osteocyte morphology and canaliculi were assessed by Ploton-Silver staining (*n* = 4/group). Patients without aluminum showed higher PTH and total alkaline phosphatase, greater osteoid volume (*p* = .0214) and fibrosis (*p* = .0251), whereas those with aluminum had higher phosphorus. Globally, OPN was higher in aluminum-associated osteomalacia (*p* = .0334), whereas RANKL (*p* = .0164) and CD44 (*p* < .0001) were higher without aluminum. In mineralized bone only CD44 differed (*p* < .0001); in osteoid, fibroblast growth factor 23 (FGF23), matrix extracellular phosphoglycoprotein (MEPE), CD44, and E11 were higher without aluminum (all *p* < .05). Patients without aluminum also showed more canaliculi per osteocyte (16 vs 14, *p* = .0468), with unchanged mean canalicular length (*p* = .113) and more slender osteocyte bodies. After adjustment for PTH, alkaline phosphatase, and phosphorus, aluminum status remained associated with CD44 globally (*p* = .0016) and in mineralized bone (*p* = .0041), and with osteoid FGF23 (*p* = .0171) and MEPE (*p* = .0468), but not with RANKL, OPN or osteoid E11. Our findings suggest that osteomalacia and aluminum-associated osteomalacia represent distinct variants, with aluminum potentially affecting osteocyte function, the canalicular network, and OPN expression. Part of the differences may nevertheless reflect the distinct biochemical milieu of the two groups.

## Graphical Abstract

**Graphical Abstract:**
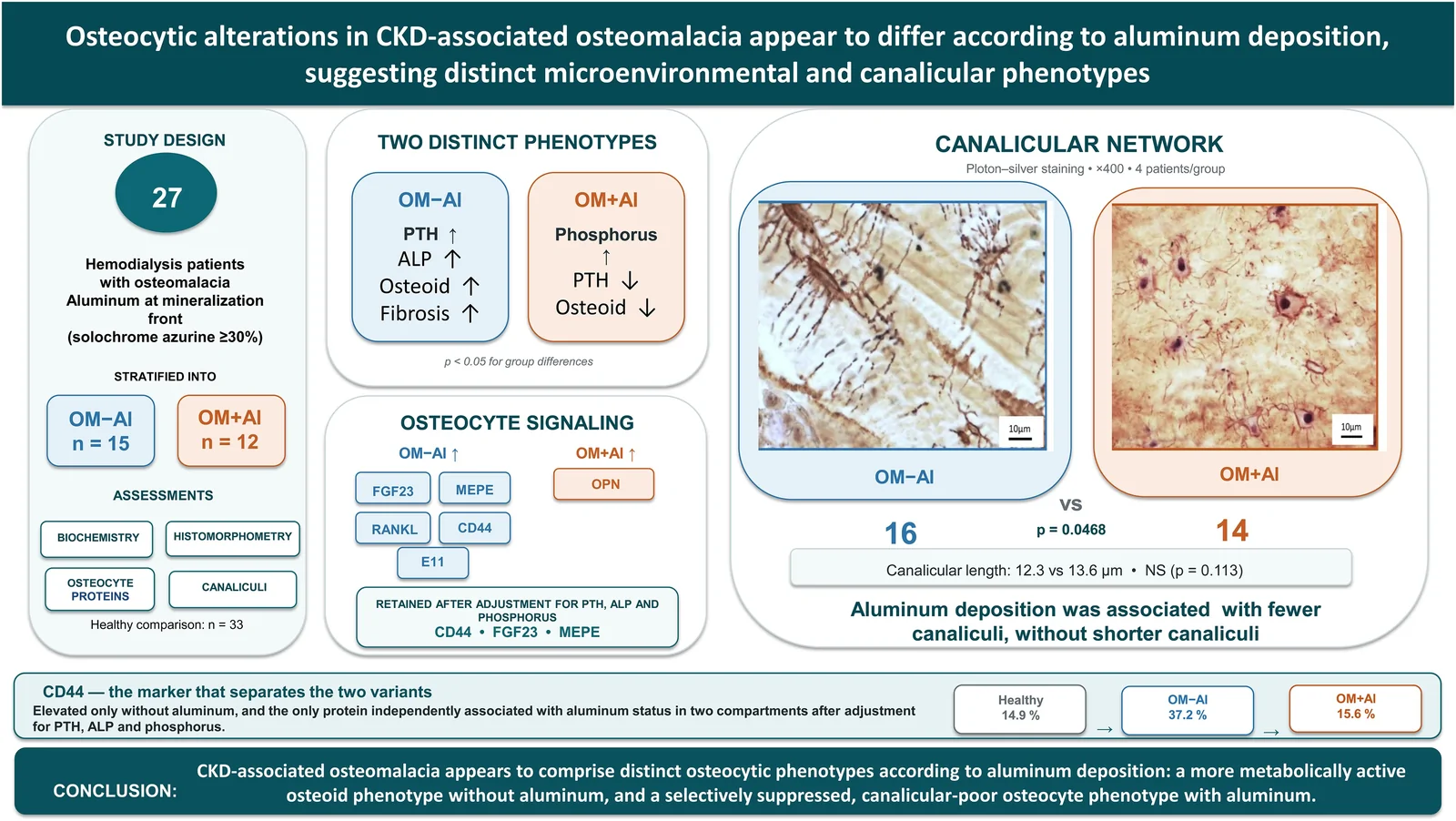

## Introduction

Osteomalacia (OM) is a severe defect of bone mineralization characterized by excessive osteoid accumulation and delayed mineral apposition.^1^ In patients with CKD, particularly those undergoing hemodialysis (HD), disturbances in mineral metabolism impair bone mineralization, and contribute to the development of renal osteodystrophy (ROD).^2^ Osteomalacia is estimated to account for approximately 5%-10% of ROD cases and may occur either in association with aluminum (Al) toxicity or in its absence.^3–5^

Historically, Al deposition at mineralization fronts has been strongly associated with impaired mineralization and low bone turnover in CKD-associated OM. Experimental and clinical studies have demonstrated that Al may interfere with osteoblast activity, matrix mineralization, and bone formation.^6^^,^^7^ However, most previous investigations focused predominantly on histomorphometric and biochemical abnormalities, whereas the cellular and microstructural alterations associated with Al deposition remain incompletely understood. Whether OM with Al deposition (OM+Al) and without Al deposition (OM-Al) represent biologically distinct skeletal phenotypes has not been fully elucidated.

Recent advances in bone biology have highlighted the central role of osteocytes in skeletal homeostasis. Beyond their classical mechanosensory function, osteocytes actively regulate bone remodeling and mineralization through the production of proteins involved in phosphate metabolism, osteoblast and osteoclast signaling, and extracellular matrix maturation.^8^^,^^9^ Disruptions in osteocyte-derived regulatory networks and abnormalities in their developmental stages are now recognized as key contributors to the development of mineral and bone disorders in CKD-MBD.^10^^,^^11^ Nevertheless, the role of osteocytes in CKD-associated OM remains poorly explored.

In addition to osteocyte-related signaling, the integrity of the lacuno-canalicular network is increasingly recognized as a critical component of bone quality and mineralization. Structural alterations of osteocyte morphology and canalicular organization may impair cellular communication and local mineralization processes.^12^^,^^13^ However, the lacuno-canalicular network has not been systematically investigated in CKD-associated OM, particularly according to the presence or absence of Al deposition.

Furthermore, bone mineralization occurs within spatially distinct microenvironments, including osteoid and mineralized matrix compartments, which may exhibit different osteocyte phenotypes and protein expression profiles.^9^^,^^14^ Compartment-specific evaluation of osteocyte-related proteins may therefore provide additional insight into the mechanisms underlying impaired mineralization in CKD-associated OM.

The present study aimed to compare clinical, biochemical, histomorphometric, and osteocyte-related alterations in HD patients with CKD-associated OM stratified according to the presence or absence of Al deposition. In addition, we evaluated osteocyte-related protein expression according to bone compartment, assessed osteopontin (OPN) expression, and performed quantitative and morphological analyses of osteocyte canaliculi.

## Patients and methods

This was a cross-section, observational, study conducted through an active search of the bone biopsy database of the Renal Pathophysiology Laboratory (LIM-16) at the Hospital das Clínicas, University of São Paulo School of Medicine, from 2000 to 2013, and of Registro Brasileiro de Biópsias ósseas (REBRABO) from 2014 to 2024. The search identified patients with CKD who had undergone bone biopsy for diagnostic evaluation of ROD. Patients with a histological diagnosis of OM, with (OM+Al) or without Al (OM-Al) deposition at bone mineralization fronts, were selected for analysis.

Al deposition was assessed histochemically in undecalcified sections stained with solochrome azurine (pH 5.0) and was evaluated along the bone mineralization front. Patients were classified as OM+Al when stainable Al was present in ≥30% of the mineralization front, according to the standardized histomorphometric criterion adopted in our laboratory,^15^ whereas patients with no detectable Al staining were classified as OM-Al. Bone biopsy staining was the only criterion used for Al classification, and the extent of deposition was quantified as the aluminum surface (Al.S/BS). Water and dialysate Al concentrations and desferrioxamine challenge test results were not systematically available throughout the whole study period (2000-2024).

Inclusion criteria were age >18 yr, both sexes, and maintenance HD for at least 6 mo. Exclusion criteria were CKD patients on conservative treatment, those undergoing peritoneal dialysis, kidney transplant recipients, patients with prior parathyroidectomy, and biopsies with insufficient technical quality that precluded reliable histomorphometric analysis.

### Biochemical assessment

Biochemical evaluation included serum measurements of total calcium (reference range: 8.4-10.2 mg/dL), phosphorus (reference range: 2.5-4.5 mg/dL), alkaline phosphatase (ALP; reference range: 40-129 U/L), PTH (reference range: 15-65 pg/mL), and 25OHD (reference range: >30 ng/mL]. All analyses were performed in a certified laboratory using standardized automated methods, as previously described in other studies.

### Bone biopsy

Transiliac crest bone biopsies were obtained using a 7-mm internal diameter trephine under local anesthesia (2% lidocaine) and intramuscular midazolam sedation. All patients underwent double tetracycline labeling (500 mg every 12 h for 3 d, separated by a 10-d drug-free interval), and biopsies were performed 3-5 d after the second course.

Undecalcified specimens were embedded in methyl methacrylate, and 5- and 10-μm sections were obtained using a HistoCore Autocut microtome (Leica Biosystems). 0.1% toluidine blue (pH 6.4) staining on 5-μm sections were used for static histomorphometric and osteocyte analyses (total number), whereas unstained 10-μm sections were evaluated under ultraviolet light for dynamic parameters.

All analyses were performed using a fluorescence microscope (Labophot 2A, Nikon) coupled to a digitizing image-analysis system (OsteoMeasure version 3.00, Osteometrics Inc.). Trabecular bone was analyzed throughout the entire biopsy specimen, excluding the cortical compartment. Static parameters were evaluated at ×125 magnification, whereas osteocyte quantification was performed at ×250 magnification. Histomorphometric nomenclature followed American Society for Bone and Mineral Research (ASBMR) recommendations, and reference values were derived from a published meta-analysis.^16^^,^^17^

### Static parameters

Static parameters were evaluated and compared with reference values for normal bone, expressed as mean and 95% CI, as previously described.^17^ Trabecular bone microarchitecture and structure were assessed using the following parameters: Bone volume (BV/TV, %—volume occupied by the trabecular bone, mineralized or not, expressed as a percentage of the tissue volume occupied by the bone marrow and trabeculae); trabecular thickness (Tb.Th, μm—average of the trabecular thickness); trabecular separation (Tb.Sp, μm—the mean distance between trabeculae); and trabecular number (Tb.N, /mm or mm^−1^—number of bone trabeculae per millimeter of tissue evaluated).

Bone formation parameters: osteoid volume (OV/BV, %—volume of unmineralized osteoid matrix relative to bone volume), osteoid thickness (O.Th, μm—thickness of the osteoid matrix), osteoid surface (OS/BS, %—percentage of trabecular surface covered by osteoid in relation to bone surface), and osteoblast surface (Ob.S/BS, %—percentage of trabecular surface lined by osteoblasts in relation to bone surface). Bone resorption: eroded surface (ES/BS, %—percentage of trabecular surface that has resorption lacunae, with or without osteoclasts) and osteoclast surface (Oc.S/BS, %—percentage of trabecular surface covered by osteoclasts in relation to bone surface).

Osteocyte parameters: osteocyte total number (Ost, *n*—osteocyte total number present in the trabeculae), aluminum surface (Al.S/BS, %—percentage of the bone surface covered by aluminum, evaluated along the mineralization front), and fibrosis volume (Fb.V/TV, %—volume of marrow fibrous tissue relative to tissue volume).

Reference values for osteocyte parameters were derived from laboratory-established normal values obtained from 33 individuals. Reference values for structural and dynamic histomorphometric parameters were derived from a published meta-analysis of healthy individuals, as previously described.^17^

### Dynamic parameters

Dynamic parameters included mineral apposition rate (MAR), mineralizing surface/bone surface (MS/BS), bone formation rate/bone surface (BFR/BS), and mineralization lag time (Mlt). Confluent tetracycline labeling was observed in all bone biopsies, making quantification impossible to perform.

### Immunohistochemistry

Immunohistochemical analyses were performed using a previously adapted method for undecalcified methyl methacrylate-embedded bone specimens.^18^ Briefly, methyl methacrylate removal using a xylene:chloroform solution (1:1), followed by rehydration in graded ethanol and semi-decalcification in 1% acetic acid.

Fibroblast growth factor 23 (FGF23), DKK1, PHEX, and E11/podoplanin were detected using the VECTASTAIN ABC-HRP system (Vector Laboratories). Sections were treated with hydrogen peroxide at 3% in methyl alcohol to block the endogenous peroxidase and avidin/biotin blocking steps.^18^^,^^19^ Briefly, depending on the antibody, nonspecific binding was blocked using either Protein Block Solution (Thermo Fisher Scientific) or normal horse serum/BSA. Primary antibodies included anti-FGF23 (1:200), anti-DKK1 (1:80), anti-PHEX (1:600), and anti-E11/podoplanin (1:50), incubated overnight at 4 °C in a humid chamber. Corresponding biotinylated secondary antibodies and avidin-biotin-peroxidase complexes were subsequently applied according to the manufacturer’s instructions.

Sclerostin, CD44, and OPN immunostaining were performed using the Reveal Polyvalent HRP system (Abcam). After endogenous peroxidase blockade and protein blocking, sections were incubated with anti-sclerostin (1:1500), anti-OPN (1:2000), or anti-CD44 (1:200) antibodies. Secondary detection steps followed the manufacturer’s recommendations.

RANKL, DMP1, OPG, and matrix extracellular phosphoglycoprotein (MEPE) were detected using the EnVision+ HRP polymer system (Dako/Agilent). After peroxidase blockade and casein blocking, sections were incubated with primary antibodies against RANKL (1:100), DMP1 (1:1000), OPG (1:800), and MEPE (1:800). Species-specific HRP-conjugated EnVision polymers were then applied according to the manufacturer’s instructions.

All incubation steps were interspersed with phosphate-buffered saline washes, and all reactions were performed under standardized conditions. Negative controls were processed by omitting the primary antibody.

Osteocytic protein expression was evaluated separately in mineralized bone and osteoid matrix compartments. Quantification was performed at ×400 magnification and expressed as the percentage of positive osteocytes relative to total osteocytes.

Osteopontin expression was analyzed throughout the entire biopsy specimen, excluding the cortical compartment, using a point-counting grid at ×20 magnification. Results were expressed as the percentage of OPN-positive area relative to total tissue area.

Apoptosis was assessed by cleaved Caspase-3 immunostaining using the SignalStain Apoptosis Detection Kit (Cell Signaling Technology). Positive cells were identified by reddish-brown nuclear staining under light microscopy at ×400 magnification and expressed as the percentage of positive osteocytes relative to total osteocytes.

For comparison with healthy individuals, immunohistochemical data from 33 healthy individuals available in our laboratory database were retrieved; this was the same group used to define normal osteocyte values. In this group, osteocyte-related proteins had been quantified in the global analysis using the same antibodies, protocols, and quantification criteria described above. Compartment-specific (mineralized bone and osteoid) quantification and serum biochemistry were not available for these individuals, so neither compartmental comparisons nor correlation analyses could be performed for the healthy group.

These healthy individuals belong to a bank of bone samples from donors maintained by our laboratory; all died from trauma or accidental causes and none had known kidney or metabolic bone disease. Their mean age was 38.0 ± 10.7 yr (range: 18-59 yr), 22 (66.7%) were women and 22 of the 32 with recorded information (68.8%) were White. The number of healthy individuals evaluated varied between antibodies according to the material available for each reaction.

### Measurable canaliculi and modified Ploton staining protocol

For each studied group, four cases were subjected to a modification of the traditional Ploton staining method,^20^^,^^21^ which is detailed below. After removal of methyl methacrylate and partial decalcification (as previously described for immunohistochemistry), the slides containing bone tissue fragments were incubated in a silver nitrate solution (0.67 M, 0.6% type B gelatin (Bloom 100), and 0.3% formic acid). After 90 min, the excess solution was removed, and the slides were washed with distilled water (two 5-min baths) and incubated for 10 min in cresyl violet solution (0.05%, pH 3.5) for nuclear staining. The slides then underwent 2 baths in distilled water, and were mounted with coverslips and Kaiser’s gelatin.

Images of trabecular osteocytes stained by the modified Ploton method, were acquired using a DS-Fi1 digital camera coupled to an ECLIPSE 50i optical microscope (Nikon Instruments Inc.) at a final magnification of 400× for canaliculi length measurements. Measurements were taken using the Measurements tool of the Image-Pro Plus vs 7.0 software (Media Cybernetics Inc.), and the values were expressed in micrometers (μm). For each case, 6-40 osteocytes were selected based on the clear visualization of both cell bodies and nuclei. For every selected osteocyte, the number of canaliculi emanating from the cell body was counted and the length of each canaliculus was measured, from which the mean canalicular length per osteocyte was derived. A total of 168 osteocytes from 4 OM-Al patients and 105 osteocytes from 4 OM+Al patients were analyzed, and the individual osteocyte was used as the unit of analysis. Osteocyte body shape and canalicular distribution were additionally assessed qualitatively by a single observer blinded to group assignment; inter-observer agreement was not evaluated.

### Statistical analysis

Statistical analyses were performed using GraphPad Prism (version 10.6.1, GraphPad Software), with significance set at *p* < .05. Normality was assessed using the Shapiro–Wilk and D’Agostino–Pearson tests. Normally distributed variables were expressed as mean ± SD and compared using the unpaired *t-*test with Welch’s correction, while non-normally distributed variables were expressed as median (minimum–maximum) and compared using the Mann–Whitney test. Categorical variables were expressed as frequencies (*n*, %) and compared using Fisher’s exact test or the chi-square test.

The association between serum PTH and each osteocyte-related parameter was assessed by Spearman rank correlation, pooling both groups in order to preserve statistical power. To determine whether the between-group differences were independent of the main biochemical confounders, multivariable linear regression models were fitted for every protein that differed significantly between groups, both globally and by compartment, including Al status, PTH, ALP, and serum phosphorus as covariates; results are reported as adjusted β coefficients with 95% CIs and model R.^2^ The relationship between RANKL expression and marrow fibrosis was assessed using Spearman correlation with Fb.V/TV, comparison of RANKL expression between biopsies with and without fibrosis (Fb.V/TV > 0), and inclusion of Fb.V/TV as a covariate in the multivariable model for global RANKL expression.

Global protein expression in the healthy individuals, OM-Al and OM+Al was compared using the Kruskal–Wallis test. When the Kruskal–Wallis test was significant, pairwise comparisons between the three groups were performed with Dunn’s post-hoc test with Holm adjustment for the three comparisons within each parameter. Because these analyses were exploratory and hypothesis-generating, no correction for multiple comparisons was applied.

## Results

A total of 27 patients with CKD-associated OM were included, comprising 15 patients OM-Al and 12 OM+Al. Clinical, laboratory, and therapeutic characteristics are presented in Table 1. The mean age was similar between groups (45.67 ± 14.97 yr in OM-Al vs 50.8 ± 13.4 yr in OM + Al; *p* = .3611). Sex distribution was similar between groups (46.6% in OM-Al vs 58.3% in OM + Al; *p* = .7036), and time on HD [66 (6-168) vs 72 (12-180) mo; *p* = .5756]. The etiology of CKD was similar between groups, with hypertension (SAH) (13.3% OM-Al vs 25% OM+Al), diabetes mellitus (DM) (33.3% OM-Al vs 8.3% OM+Al), and tubulopathies (13.3% OM vs 16.6% OM+Al) being the main recorded causes. A substantial proportion of patients had CKD of unknown etiology (40% OM-Al vs 50% OM+Al). No differences were observed between groups regarding clinical signs, symptoms, and medications.

**Table 1:** Clinical, laboratory, and medication characteristics of patients with OM-Al and OM+Al.

Parameters	OM-Al (*n* = 15)	OM + Al (*n* = 12)	*p*
**Clinical**			
**Age (yr)**	45.67 ± 14.97	50.75 ± 13.37	.3611^a^
**Male sex (*n*)**	7 (46.6%)	7 (58.3%)	.7036^c^
**Ethnicity (White) (*n*)**	6 (40%)	3 (25%)	.6828^c^
**CKD etiology (*n*)**			
**Hypertension**	2 (13.3%)	3 (25%)	.5081^c^
**Diabetes mellitus**	5 (33.3%)	1 (8.3%)	
**Tubulopathy**	2 (13.3%)	2 (16.6%)	
**Undetermined**	6 (40%)	6 (50%)	
**HD duration (mo)**	66.00 (6-168)	72 (12-180)	.5756
**Fractures (*n*)**	7 (50%) (*n* = 14)	4 (45%) (*n* = 9)	.9999^c^
**Pain (*n*)**	12 (80%)	10 (83.3%)	.9999^c^
**Myalgia (*n*)**	8 (53.3%)	5 (71.4%) (*n* = 7)	.7036^c^
**Muscle weakness (*n*)**	11 (73.3%)	3 (75%) (*n* = 4)	.9999^c^
**Deformities**	2 (25%)	4 (44.4%)	.6199^c^
**Medications**			
**Calcium carbonate (*n*)**	4 (26%)	4 (33%)	.9999^c^
**Sevelamer (*n*)**	6 (40%)	1 (8%)	.0914^c^
**Vitamin D analogs (*n*)**	8 (53%)	3 (25%)	.2388^c^
**Cinacalcet (*n*)**	1 (6.6%)	0	.9999^c^
**Colecalciferol (*n*)**	1 (6.6%)	3 (25%)	.2940^c^
**Biochemical**			
**Albumin (mg/dL)**	3.7 (3.4-3.9) (*n* = 4)	3.85 (3.3-4.1)	.8180^b^
**Total calcium (mg/dL)**	8.8 (7-10.1)	9.25 (6.8-10.7)	.2414^b^
**Phosphorus (mg/dL)**	**3.1 (1.3-4.4)**	**4.27 (1-7.2)**	**.0340^b^**
**ALP (U/L)**	**220 (51-590)**	**120 (41-439)**	**.0154^b^**
**PTH (pg/mL)**	**287 (60-1101)**	**47.65 (10-396)**	**.0120^b^**

a Student’s *t-*test.

b Mann–Whitney test.

c Fisher’s exact test.

Data are presented as mean ± SD, median (minimum–maximum), or *n* (%), as appropriate. Values in bold indicate statistical significance at *p* < .05. Abbreviations: ALP, alkaline phosphatase; CaT, total calcium; HD, hemodialysis; *n*, number of patients; OM-Al, patients with osteomalacia without Al deposition; OM+Al, patients with osteomalacia with aluminum deposition.

Regarding biochemical parameters, patients with OM+Al presented higher P levels compared with patients with OM-Al [4.27 (1-7.2) vs 3.1 (1.3-4.4) mg/dL; *p* = .0340]. Conversely, ALP [120 (41-439) vs 220 (51-590) U/L; *p* = .0154] and PTH levels [47.65 (10-396) vs 287 (60-1101) pg/mL; *p* = .0120] were lower in patients with OM+Al.

Histomorphometric results are summarized in Table 2. Significant differences were observed between groups in osteoid volume (OV/BV) and fibrosis volume (Fb.V/TV). Patients with OM-Al showed higher OV/BV compared with those with OM+Al (37.86 ± 16.46% vs 23.39 ± 14.74%; *p* = .0214), as well as higher Fb.V/TV (0.82 ± 1.23% vs 0.18 ± 0.53%; *p* = .0251). Aluminum (Al.S/BS, %) was absent in the OM-Al group, whereas it was detected along 100% of the mineralization front in 11 of the 12 OM+Al biopsies, and 50% of it in the remaining one.

**Table 2:** Comparison of histomorphometric parameters in patients with OM-Al and OM+Al.

Parameters	OM-Al (*n* = 15)	OM+Al (*n* = 12)	*p*	Healthy: mean (95% CI)
**Structural parameters**				
**Bone volume (BV/TV, %)**	37.8 ± 13.8	30.8 ± 10.4	.2773	20.46 (19.55-21.36)
**Trabecular thickness (Tb.Th, μm)**	128.8 ± 29.9	132.2 ± 32	.7551	143.36 (135.2-151.53)
**Trabecular separation (Tb.Sp, μm)**	425.3 ± 204.4	324.4 ± 122.2	.2363	639.1 (578-700.3)
**Trabecular number (Tb.N, /mm)**	2 ± 0.9	2.3 ± 0.9	.1634	1.5 (1.3-1.6)
**Formation parameters**				
**Osteoid volume (OV/BV, %)**	**37.8 ± 16.4**	**23.3 ± 14.7**	**.0214**	**2.22 (1.83-2.62)**
**Osteoid thickness (O.Th, μm)**	28.8 ± 12.8	20.8 ± 11	.0529	9.87 (8.83-10.91)
**Osteoid surface (OS/BS, %)**	78.7 ± 15.4	69.63 ± 25.7	.5480	14.64 (13.55-15.73)
**Osteoblastic surface (Ob.S/BS, %)**	5.7 ± 5.6	2.8 ± 2.6	.0926	3.62 (2.04-5.2)
**Resorption parameters**				
**Resorption surface (ES/BS, %)**	4.6 ± 4.6	2.4 ± 2.1	.3052	4.3 (3.67-4.93)
**Osteoclastic surface (Oc.S/BS, %)**	1.1 ± 1.3	0.5 ± 0.9	.2491	1.04 (0.6-1.49)
**Fibrosis volume (Fb.V/TV, %)**	**0.8 ± 1.2**	**0.2 ± 0.5**	**.0251**	**0**
**Aluminum (Al.S/BS, %)**	**0**	**95.8 ± 14.4**	**<.0001**	0
**Osteocytes**				
**Total osteocyte number** **(Total Ot, n)**	467 ± 335.5	454 ± 298	.9046	
**Ot. B.Md (n)**	311.2 ± 275.1	324.5 ± 203.7	.4095	
**Ot.O (n)**	100.9 ± 123.4	123.6 ± 171.7	.5512	
**Li.Ce (n)**	127.2 ± 110.3	206.4 ± 104.5	.0688	

Data are presented as mean ± SD. Values in bold indicate statistical significance at *p* < .05. Healthy values are presented as mean (95% CI). Abbreviations: Al.S/BS, aluminum surface; B.Md, mineralized bone matrix; BV/TV, bone volume; ES/BS, resorption surface; Fb.V/TV, fibrosis volume; Li.Ce, lining cells; Ob.S/BS, osteoblastic surface; Oc.S/BS, osteoclastic surface; OM-Al, patients with osteomalacia without Al deposition; OM+Al, patients with osteomalacia with aluminum deposition; O.Th, osteoid thickness; OS/BS, osteoid surface; Ot.O, osteocytes in osteoid; OV/BV, osteoid volume; Tb.N, trabecular number; Tb.Sp, trabecular separation; Tb.Th, trabecular thickness; Total Ot, total osteocyte number.

Comparison of osteocytic protein expression, OPN expression, and osteocyte apoptosis between patients with OM-Al and OM+Al, stratified according to their functions related to bone remodeling, mineralization, and cytoskeletal organization, is presented in Table 3. Patients with OM-Al exhibited higher RANKL expression compared with the OM+Al group [4.4 (0-40) vs 2.35 (0-9.4); *p* = .0164], as well as higher CD44 expression [37.22 (28.79-72.75) vs 15.64 (10.79-26.17); *p* < .0001]. In contrast, OPN expression was significantly higher in the OM+Al group [57.4 (34-66) vs 40.9 (30-63.2); *p* = .0334] Representative immunohistochemical staining for RANKL, CD44 and OPN in trabecular bone from patients with OM-Al and OM+Al is shown in Figure 1.

**Table 3:** Comparison of osteocytic protein expression, OPN, and apoptosis in the global analysis between patients with OM-Al and OM+Al.

	OM-Al (*n* = 15)	OM+Al (*n* = 12)	*p*
**Remodeling**			
**Scl (%)**	3.3 (0-18.3)	2.9 (1-27.5)	.5392
**RANKL (%)**	**4.4 (0**-**40)**	**2.35 (0**-**9.4)**	**.0164**
**OPG (%)**	8.5 (0-43.2)	5 (3-32.8)	.6831
**DKK1 (%)**	1.3 (0-12)	0.95 (0-13.5)	.7934
**Mineralization**			
**DMP1 (%)**	6 (0.94-23.2)	7.65 (0.7-44.9)	.4061
**FGF23 (%)**	3.06 (0-17.25)	2.75 (0-10.81)	.4548
**MEPE (%)**	9.16 (1.5-53.7)	8 (3.6-31.7)	.9046
**PHEX (%)**	7.85 (1.3-36)	11 (1-22.4)	.4199
**OPN (%)**	**40.9 (30**-**63.2)**	**57.4 (34**-**66)**	**.0334**
**Cytoskeleton**			
**CD44 (%)**	**37.22 (28.79**-**72.75)**	**15.64 (10.79-26.17)**	**<.0001**
**E11 (%)**	9.7 (4.2-53.7)	9.2 (3.1-34)	.8014
**Apoptosis (%)**	0.8 (0-13.2)	0.41 (0-8.11)	.7957

Data are presented as median (minimum–maximum). Values in bold indicate statistical significance at *p* < .05. Comparisons between groups were performed using the Mann–Whitney test. Abbreviations: DKK1, Dickkopf-1 protein; DMP1, dentin matrix protein 1; E11, podoplanin; FGF23, fibroblast growth factor 23; MEPE, matrix extracellular phosphoglycoprotein; OM-Al, patients with osteomalacia without Al deposition; OM+Al, patients with osteomalacia with aluminum deposition; OPG, osteoprotegerin; OPN, osteopontin; PHEX, phosphate-regulating neutral endopeptidase; Scl, sclerostin.

**Figure 1:**
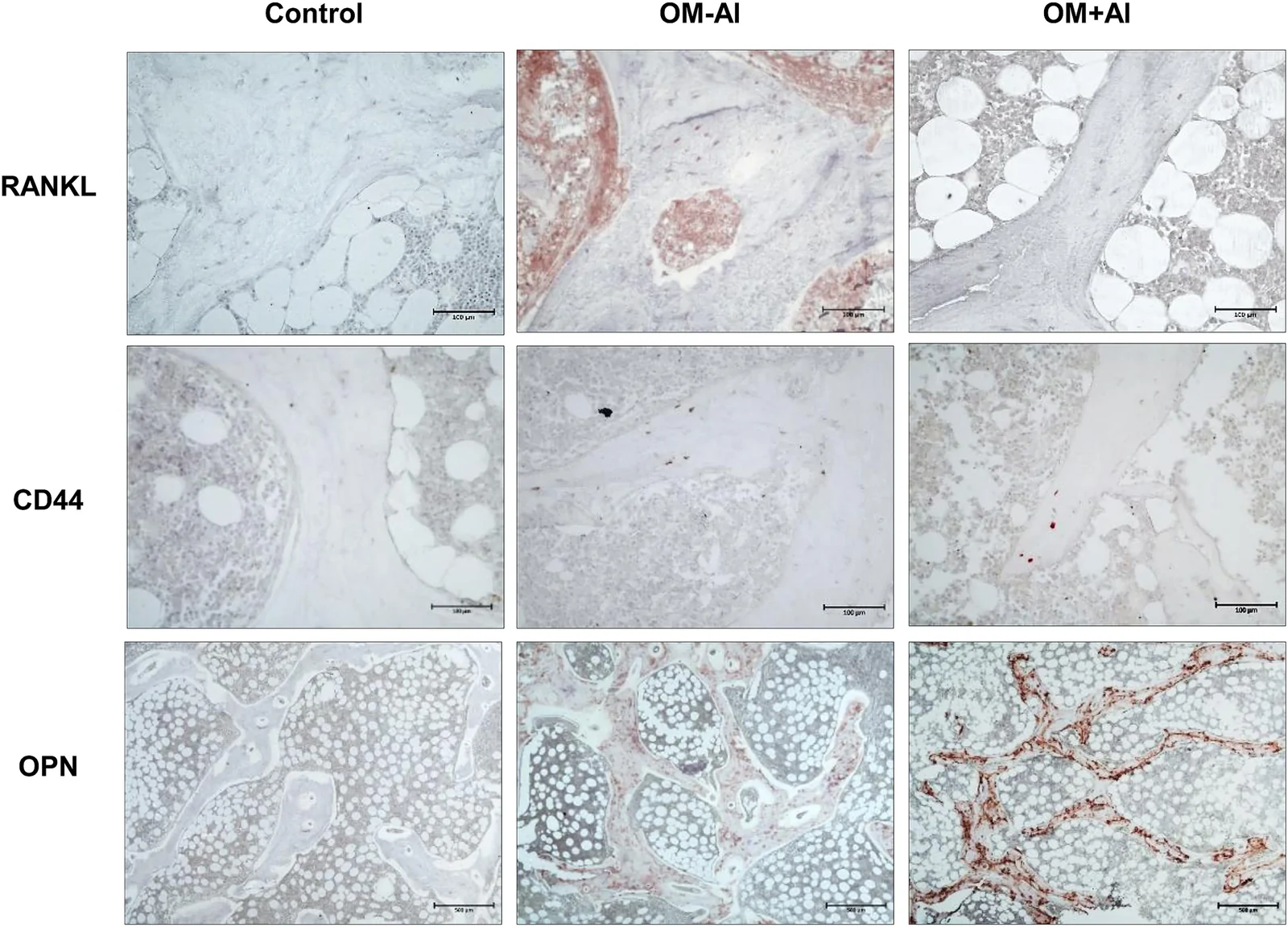
Representative microscopic features of osteocyte protein expression by immunohistochemistry. Representative immunostaining for RANKL and CD44 (original magnification ×200) and OPN (original magnification ×40) in bone tissue from patients with OM-Al, with OM+Al and negative control of reaction are also shown. Increased RANKL and CD44 immunostaining was observed in OM-Al, whereas OPN staining was more prominent in OM+Al. OM-Al, osteomalacia without aluminum deposition; OM+Al, osteomalacia with aluminum deposition. Scale bars = 100 μm.

The expression of osteocyte related proteins in mineralized bone is presented in Table 4. Among the proteins evaluated, only CD44 expression differed significantly between groups. CD44 expression was higher in patients with OM-Al compared with OM+Al [39.85 (30.9-83.2) vs 17.9 (4.4-31.7); *p* < .0001]. No significant differences between groups were observed for other remodeling markers (Scl, RANKL, OPG, and DKK1), mineralization-related proteins (DMP1, FGF23, MEPE, and PHEX), or E11.

**Table 4:** Comparison of osteocytic protein expression between patients with OM-Al and OM+Al in mineralized bone matrix.

	OM-Al (*n* = 15)	OM+Al (*n* = 12)	*p*
**Remodeling**			
Scl.B.Md **(%)**	4.5 (0-22)	3.5 (1.7-30.5)	.9881
RANKL.B.Md **(%)**	4.2 (0-89.5)	2.2 (0-8.1)	.0607
OPG.B.Md **(%)**	6.1 (1.2-29.2)	5 (1.6-24.5)	.8536
DKK1.B.Md **(%)**	1.6 (0-10.5)	0.6 (0-15)	.5226
**Mineralization**			
DMP1.B.Md **(%)**	10 (2.4-51.6)	5.8 (0-47.5)	.3255
FGF23.B.Md **(%)**	2 (0-17.4)	2.3 (0-12)	.9894
MEPE.B.Md **(%)**	11.1 (0-36.1)	10.6 (3.2-36.8)	.6297
PHEX.B.Md **(%)**	5.5 (1-46.9)	10.1 (1.2-30.5)	.2415
**Cytoskeleton**			
CD44.B.Md **(%)**	**40 (30.9-83.2)**	**18 (4.4-31.7)**	**<.0001**
E11.B.Md **(%)**	9.1 (0-20.1)	10.2 (3-24.6)	.3190

Data are presented as median (minimum–maximum). Values in bold indicate statistical significance at *p* < .05. Comparisons between groups were performed using the Mann–Whitney test. Abbreviations: B.Md, mineralized bone matrix; DKK1, Dickkopf-1 protein; DMP1, dentin matrix protein 1; E11, podoplanin; FGF23, fibroblast growth factor 23; MEPE, matrix extracellular phosphoglycoprotein; OM, osteomalacia; OM-Al, patients with osteomalacia without Al deposition; OM+Al, patients with osteomalacia with aluminum deposition; OPG, osteoprotegerin; PHEX, phosphate-regulating neutral endopeptidase; Scl, sclerostin.

Protein expression in the osteoid matrix compartment is presented in Table 5. Significant differences were observed for FGF23, MEPE, CD44, and E11, all showing higher expression in the OM-Al group compared with OM+Al. Fibroblast growth factor 23 expression was higher in OM-Al [7.5 (0-56)] compared with OM+Al [0 (0-8); *p* = .0329]. Similarly, MEPE [15.15 (0-55.6) vs 2.7 (0-20); *p* = .0110], CD44 [21 (0-59) vs 5 (0-81); *p* = .0205], and E11 [26.2 (0-86.2) vs 5.25 (0-66.7); *p* = .0423] were significantly higher in the OM-Al group. No differences were observed for other evaluated proteins.

**Table 5:** Comparison of osteocytic protein expression between patients with OM-Al and OM+Al in the osteoid matrix.

	OM-Al (*n* = 15)	OM+Al (*n* = 12)	*p*
**Remodeling**			
**Scl.O (%)**	0.7 (0-66.7)	0 (0-5.3)	.1131
**RANKL.O (%)**	10.10 (0-33.3)	0.2 (0-19.4)	.0615
**OPG.O (%)**	18.9 (0-89.7)	10.95 (0-66.7)	.1828
**DKK1.O (%)**	0 (0-17.2)	0	.4783
**Mineralization**			
**DMP1.O (%)**	0 (0-23.3)	0 (0-13.4)	.8953
**FGF23.O (%)**	**7.5 (0**-**56)**	**0 (0**-**8)**	**.0329**
**MEPE.O (%)**	**15.15 (0**-**55.6)**	**2.7 (0**-**20)**	**.0110**
**PHEX.O (%)**	5.75 (0-22.9)	0.2 (0-40)	.6119
**Cytoskeleton**			
**CD44.O (%)**	**21 (0**-**59)**	**5 (0-81)**	**.0205**
**E11.O (%)**	**26.2 (0**-**86.2)**	**5.25 (0**-**66.7)**	**.0423**

Data are presented as median (minimum–maximum). Values in bold indicate statistical significance at *p* < .05. Comparisons between groups were performed using the Mann–Whitney test. Abbreviations: DKK1, Dickkopf-1 protein; DMP1, dentin matrix protein 1; E11, podoplanin; FGF23, fibroblast growth factor 23; MEPE, matrix extracellular phosphoglycoprotein; O, osteoid matrix; OM, osteomalacia; OM Al+, osteomalacia with aluminum deposition; OM-Al, patients with osteomalacia without Al deposition; OM+Al, patients with osteomalacia with aluminum deposition; OPG, osteoprotegerin; PHEX, phosphate-regulating neutral endopeptidase; Scl, sclerostin.

The distribution of the individual patient values underlying Tables 3–5 is shown in Figure S1 (global analysis), Figure S2 (mineralized bone), and Figure S3 (osteoid), where individual data points are overlaid on box-and-whisker plots.

Because PTH values ranged widely within both groups, Spearman correlations were performed between serum PTH and every osteocyte-related parameter in the 26 patients with available PTH data. Significant correlations were found for global CD44 (*r* = 0.522, *p* = .0062), global FGF23 (*r* = −0.397, *p* = .0446), global osteocyte apoptosis (*r* = −0.462, *p* = .0174), CD44 in mineralized bone (*r* = 0.428, *p* = .0416), and MEPE in osteoid (*r* = 0.417, *p* = .0475); all remaining correlations were non-significant (*p* > .1) (Figure S4).

In multivariable linear regression models including Al status, PTH, ALP and P, Al status remained independently associated with global CD44 (β = +18.64; 95% CI: 8.03-29.24; *p* = .0016; *R*^2^ = 0.74), CD44 in mineralized bone (β = +33.01; 95% CI: 12.08-53.94; *p* = .0041; *R*^2^ = 0.63), FGF23 in osteoid (β = +17.17; 95% CI: 3.49-30.85; *p* = .0171; *R*^2^ = 0.50), and MEPE in osteoid (β = +20.40; 95% CI: 0.33-40.48; *p* = .0468; *R*^2^ = 0.39). Conversely, the differences in global RANKL, global OPN, CD44 in osteoid and E11 in osteoid were no longer significant after adjustment (all *p* ≥ .10) (Table S1). Positive β values indicate higher expression in OM-Al.

Marrow fibrosis was present in 12 of 15 OM-Al and 5 of 12 OM+Al biopsies. Osteoid RANKL was higher in biopsies containing any fibrosis than in those without [9.55 (0-26.5) vs 0 (0-33.3); *p* = .0170], whereas global RANKL showed only a non-significant trend with Fb.V/TV (*r* = 0.336; *p* = .0865) and no correlation within OM-Al alone (*r* = 0.158; *p* = .5732). Adding Fb.V/TV to the model for global RANKL left the Al-status coefficient unchanged (β = +8.41; 95% CI: −2.19 to 19.01; *p* = .1127), with no contribution of fibrosis (β = +0.80; *p* = .6884).

To place these findings in context, global protein expression was compared with that of the healthy individuals (*n* = 33). Seven of the 12 parameters differed significantly across the 3 groups: RANKL (*p* = .0326), CD44 (*p* < .0001), OPG (*p* = .0021), E11 (*p* < .0001), PHEX (*p* = .0033), OPN (*p* < .0001), and osteocyte apoptosis (*p* = .0051), whereas DMP1, FGF23, sclerostin, MEPE, and DKK1 did not (all *p* > .05). CD44 was elevated only in OM-Al (37.22%) compared with both the healthy individuals (14.90%) and OM+Al (15.63%), whereas OPG, E11, PHEX, OPN, and apoptosis were elevated in both OM groups relative to the healthy individuals, irrespective of Al status. RANKL was lower in OM+Al than in the healthy individuals, while OM-Al showed values close to healthy ones (Figure 3 and Table S2).

Canalicular characteristics were also evaluated regarding the number of canaliculi per osteocyte, as well as qualitative features of osteocyte body and canalicular morphology. The OM+Al group exhibited more rounded osteocyte bodies and more irregularly distributed canaliculi, whereas osteocytes in the OM-Al group appeared more elongated and regularly distributed, as illustrated in Figure 2. Quantitative analysis demonstrated a lower number of canaliculi per osteocyte in the OM+Al group (median = 14; *n* = 105) compared with the OM-Al group (median = 16; *n* = 168) (*p* = .0468) (Figure 2E). These analyses were performed at the individual osteocyte level (168 osteocytes from 4 OM-Al patients and 105 osteocytes from 4 OM+Al patients). In contrast, the mean canalicular length per osteocyte did not differ between groups (*p* = .113; Figure 2F), indicating that Al deposition was associated primarily with a reduction in canalicular density rather than in canalicular length.

**Figure 2:**
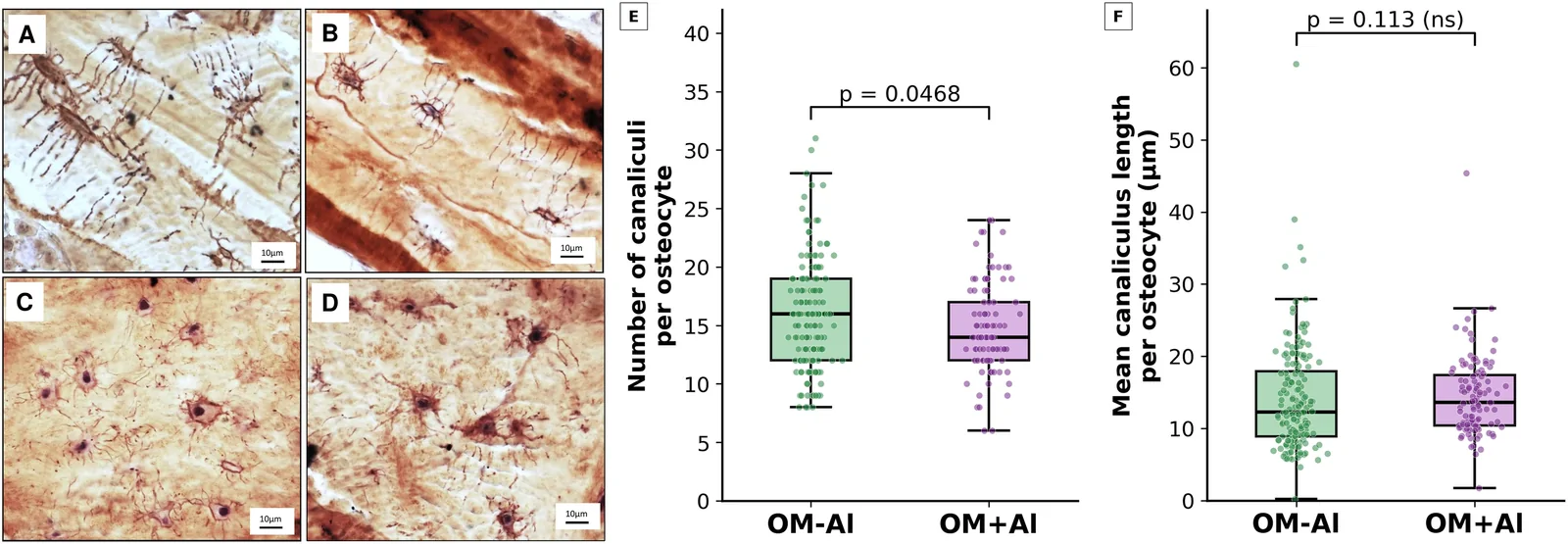
Osteocyte morphology and osteocyte canaliculi number in patients with OM-Al and OM+Al. (A and B) Representative images of osteocyte body morphology and canaliculi in trabecular bone from patients with OM-Al. (C and D) Representative images of osteocyte body morphology and canaliculi in trabecular bone from patients with OM+Al. Bone samples were stained with silver nitrate using the Ploton method (original magnification ×400). (E) Number of canaliculi per osteocyte and (F) mean canalicular length per osteocyte (μm) in OM-Al and OM+Al. Box-and-whisker plots show the median and interquartile range, with each dot representing one osteocyte (168 osteocytes from 4 OM-Al patients and 105 osteocytes from 4 OM+Al patients); the individual osteocyte was the unit of analysis. Statistical analysis was performed using the Mann–Whitney test. OM-Al, osteomalacia without aluminum deposition; OM+Al, osteomalacia with aluminum deposition. Scale bars = 10 μm.

**Figure 3:**
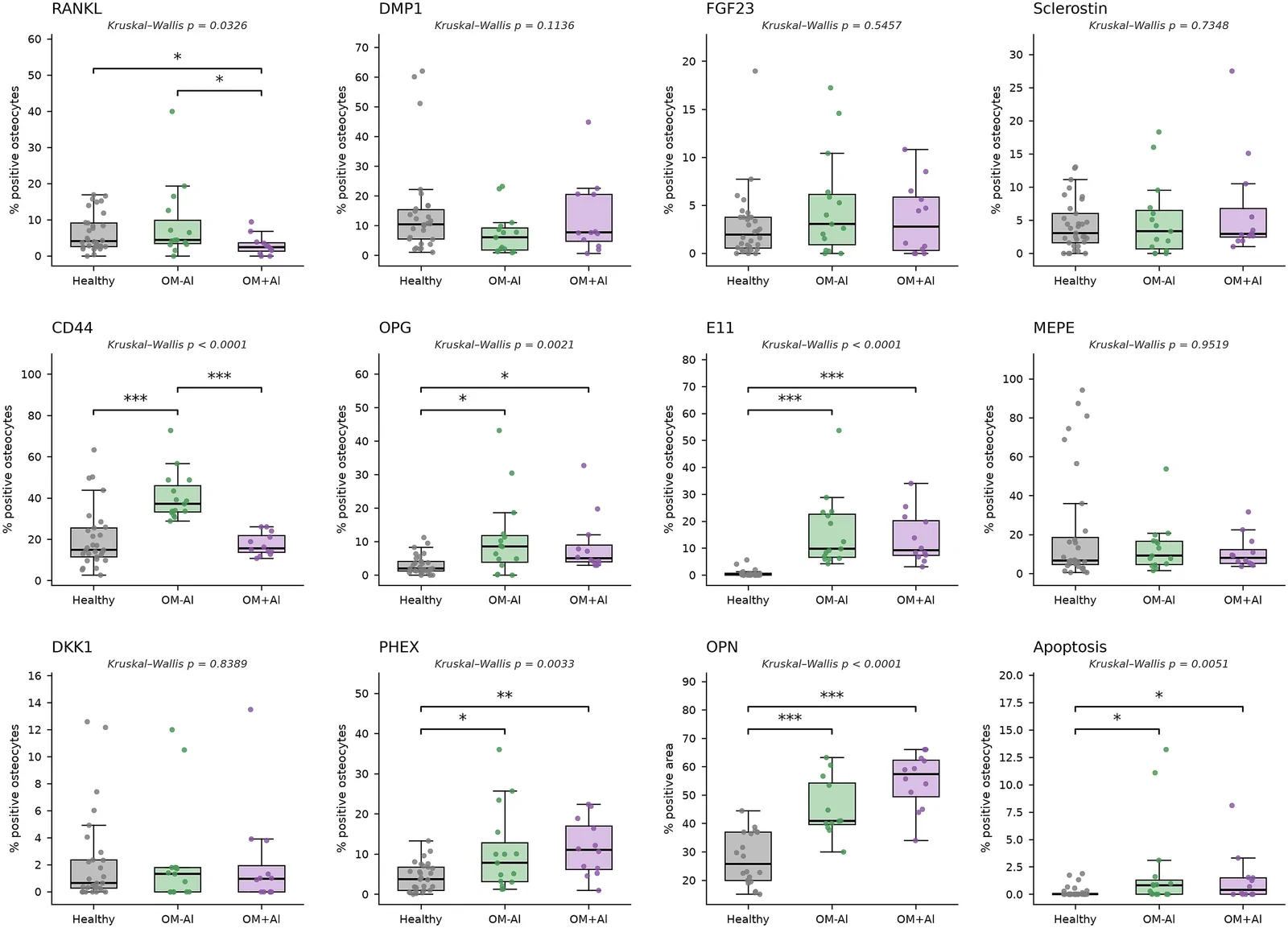
Osteocyte-related protein expression and apoptosis in healthy individuals, OM-Al and OM+Al. OM-Al, osteomalacia without aluminum deposition; OM+Al: osteomalacia with aluminum deposition. Box-and-whisker plots of the twelve parameters evaluated in the global analysis (sclerostin, RANKL, OPG, DKK1, DMP1, FGF23, MEPE, PHEX, OPN, CD44, E11, and osteocyte apoptosis), expressed as the percentage of positive osteocytes, except OPN (percentage of OPN-positive area). Each dot represents one individual and the number of healthy individuals varies between parameters (*n* = 20-33). The 3 groups were compared with the Kruskal–Wallis test, whose *p*-value is shown above each panel. Panels without brackets had no significant pairwise difference. Brackets indicate the pairwise comparisons that reached significance in Dunn’s post-hoc test with Holm adjustment (^*^p < .05, ^**^p < .01, ^***^p < .001).

Examination of solochrome azurine-stained sections revealed no pericellular Al around osteocyte lacunae within osteoid, with staining restricted entirely to the mineralization front across all OM+Al biopsies.

## Discussion

The present study demonstrates that CKD-associated OM exhibits distinct osteocyte-related and canalicular alterations according to the presence of Al deposition. Important differences were observed in biochemical, histomorphometric, osteocytic, and structural parameters, supporting the concept that patients with OM-Al and OM+Al represent biologically distinct skeletal phenotypes rather than variations of the same mineralization defect. Interpreting these differences requires separating what may be attributed to Al itself from what follows from the markedly different hormonal and mineral milieu of the 2 groups, since PTH, ALP, and P differed between them and are themselves regulators of several of the proteins examined.

Patients with OM-Al exhibited higher serum PTH and ALP levels, as well as higher OV/BV and greater Fb.V/TV, suggesting a skeletal microenvironment characterized by greater remodeling activity than in OM+Al. Previous studies have shown that Al deposition may suppress parathyroid activity and reduce both PTH synthesis and secretion, contributing to lower bone remodeling and impaired osteoblastic activity.^22–24^ In addition, experimental evidence suggests that Al may directly inhibit osteoblast-related enzymes involved in bone formation, which may explain the lower ALP levels observed in OM+Al patients.^24^^,^^25^ Consistently, the lower OV/BV observed in OM+Al likely reflects reduced osteoblastic activity related to the toxic effects of Al, lower serum PTH levels, and low bone turnover.^15^^,^^25^^,^^26^

Because that hormonal and mineral environment is itself shaped by Al, the osteocytic findings were evaluated in the context of concurrent PTH, ALP, and *p*-values, with each bone compartment examined on its own. Osteoid is where primary mineralization takes place and where the newly embedded osteocyte still secretes into an unmineralized matrix, while mineralized bone reflects a later stage of maturation in which the mature osteocyte governs its territory through the canalicular network.^10^^,^^14^^,^^27^^,^^28^ A difference confined to osteoid points toward the active process of mineralization rather than the maintenance of established bone. Importantly, not all osteocytic differences exhibited the same relationship with Al deposition. Following multivariable adjustment for PTH, ALP, and P, CD44 (globally and in mineralized bone) as well as osteoid FGF23 and MEPE remained independently associated with Al status, whereas RANKL, OPN, and osteoid expression of CD44 and E11 lost statistical significance.

Mechanistically, the markers that retained an independent association with Al reflect essential functions operating within the osteocyte’s immediate territory: a matrix-adhesion receptor through which osteocytes anchor to their surrounding matrix (CD44),^29^ and 2 local regulators of phosphate handling in the osteoid compartment (FGF23 and MEPE).^30^^,^^31^ Conversely, the markers that lost significance after adjustment correspond to systemically regulated processes, such as remodeling control and systemic phosphate clearance, that are predominantly driven by circulating PTH and P levels.

Together, these findings indicate that the osteocytic phenotype of Al-associated OM comprises a distinct, local cellular core that cannot be explained by systemic biochemical abnormalities, alongside secondary features shaped by the circulating hormonal milieu.

The compartmental distribution of these findings strongly reinforces this interpretation. Because circulating PTH and ALP act systemically across the skeleton, changes driven exclusively by the hormonal environment should manifest uniformly across both mineralized bone and osteoid. Instead, we observed a clear compartmental dissociation: four of the 10 proteins evaluated in osteoid differed significantly between groups, whereas only CD44 differed in mineralized bone, with no marker altered exclusively in that compartment. Molecular differences confined to the newly formed, unmineralized matrix, where osteocytes actively participate in primary mineralization, do not align with what would be expected from a purely systemic hormonal effect. Furthermore, although CD44 was altered in both compartments and correlated with serum PTH (*r* = 0.522), consistent with a generalized systemic response, its persistent association with Al following multivariable adjustment demonstrates that PTH alone does not account for its upregulation.

The OPN excess of OM+Al bears directly on the mineralization defect. Given the role of OPN as an inhibitor of hydroxyapatite crystal nucleation and growth,^32^ its increased expression may further contribute to impaired mineralization in these patients. Its mineral and hormonal environment accounts for that excess. Phosphate is a specific inducer of OPN gene expression^33^ and was significantly higher in OM+Al, while PTH suppresses OPN transcription in osteoblastic cells^34^ and was markedly lower, so the stimulus to OPN was increased at the same time as the restraint upon it was relieved, and the group difference disappeared once these variables were accounted for. A direct action of the metal is unlikely, since Al decreases rather than increases OPN expression in bone-forming cells in vitro*.*^35^ Osteopontin is an inflammation-responsive protein that can be induced by cytokines, such as IL-1β and TNF-α,^36^ and Al exposure can activate inflammatory pathways,^37^ so an inflammatory contribution cannot be excluded, although it is not required here. The OPN excess of Al-associated OM thus appears to be a product of the phosphate-rich, PTH-poor milieu that Al itself creates rather than of the metal acting directly on the osteocyte.

In addition, the concomitant increase in FGF23 and MEPE expression in osteoid may reflect activation of pathways involved in phosphate handling and mineralization regulation. Since the PHEX-DMP1-MEPE axis plays a central role in extracellular matrix mineralization and regulation of FGF23 production,^31^ these findings support the presence of a biologically active osteoid microenvironment in OM-Al despite defective mineralization. The axis did not, however, behave as a unit. MEPE and FGF23 differed between groups in osteoid and remained associated with Al after adjustment, whereas DMP1 and PHEX differed in neither compartment, which argues against coordinated regulation of the axis and in favor of differential control of its components in this compartment. DMP1 is the more informative of the two null results. In mineralized bone it was readily detectable and still did not differ between groups, despite an almost 6-fold difference in serum PTH, and since PTH downregulates the mature osteocyte phenotype, including DMP1,^38^ lower values would have been expected in OM-Al. Its stability suggests that when mineralization is severely impaired, the local matrix environment constrains DMP1 expression more tightly than systemic hormonal signals do.

The higher FGF23 expression in the osteoid of OM-Al sits awkwardly with the biochemical profile of the cohort, since serum P, a potent stimulus for FGF23 production, was higher in OM+Al. Two readings are available and they are not equally supported. Osteocytes in OM+Al may fail to raise FGF23 despite the phosphate load, which would place a defect in phosphate sensing, or in the osteocytic response to it, among the consequences of Al deposition; alternatively, the much higher PTH of OM-Al could act as the dominant local stimulus. The correlation we found between serum PTH and global FGF23 expression was negative (*r* = −0.397), which does not support PTH as a stimulatory driver at tissue level, and the osteoid FGF23 difference persisted after adjustment for P and PTH. A blunted osteocytic response to phosphate in the presence of Al is therefore the reading more consistent with our data. Serum FGF23 was not measured in this retrospective cohort, so no inference about the systemic FGF23 axis can be drawn, and this interpretation remains to be tested directly.

Beyond protein expression, OM+Al exhibited structural alterations suggestive of osteocyte dysfunction. Osteocytes displayed a more rounded morphology associated with a disorganized lacuno-canalicular network and fewer canaliculi per osteocyte. Structural integrity of the lacuno-canalicular network is increasingly recognized as a critical determinant of bone quality, cellular communication, mechanotransduction, and mineral homeostasis.^12^^,^^13^ Therefore, the canalicular disorganization observed in OM+Al may represent an additional mechanism contributing to impaired mineralization. The nature of this canalicular defect is further specified by the observation that canalicular number was reduced while mean canalicular length was preserved, which points to impaired formation or branching of dendritic processes rather than to their retraction or shortening. Two cautions apply to this comparison: PTH alters osteocyte morphology and induces bone cell motility^38^ and was higher in OM-Al, while the higher phosphate concentrations of OM+Al may themselves influence cell shape, so the contrast may not be Al-specific.

Whether the canalicular changes explain the protein findings or merely accompany them cannot be settled by our design, although the alternatives are not equally consistent with the data. Osteocytes with fewer canaliculi have less membrane surface available for secretion and matrix communication, so the lower protein expression detected in osteoid could reflect a damaged secretory network rather than reduced production. Such a mechanism, being non-specific, would be expected to lower osteocytic protein detection in that compartment across the board, whereas only four of the ten proteins evaluated in osteoid differed between groups. The selectivity of the reduction thus favors regulated differences in osteocytic activity over a purely structural loss of secretory surface, without excluding a contribution from the latter.

Interestingly, no differences were observed in total osteocyte number between OM groups, either globally or after separate evaluation of mineralized bone and osteoid compartments, suggesting that the osteocytic alterations were predominantly qualitative. The comparison with the healthy individuals clarifies which of these qualitative alterations belong to OM per se rather than to Al deposition.

OPG, E11, PHEX, OPN, and osteocyte apoptosis were elevated in both OM groups relative to the healthy individuals, irrespective of Al status. E11/podoplanin is expressed by early osteocytes embedded in unmineralized matrix, and its marked elevation in both OM groups relative to the healthy individuals indicates that E11 accumulation primarily reflects the osteomalacic state, a consequence of osteocytes remaining trapped in an unmineralized matrix, while its apparent enhancement in OM-Al is largely attributable to accompanying biochemical differences. Similarly, PHEX elevation across both OM groups reflects a response to OM itself rather than to Al.

In contrast, CD44 behaved distinctly: it was elevated only in OM-Al, whereas OM+Al values approached those of the healthy individuals. Notably, CD44 was also the protein that remained independently associated with Al in 2 compartments following multivariable adjustment. Among the proteins examined, CD44 is thus the principal marker whose alteration specifically distinguishes the 2 OM phenotypes, rather than merely accompanying defective mineralization in general.

Furthermore, increased RANKL expression in OM may reflect differences in systemic bone metabolism regulation, potentially mediated by PTH and P.^39–41^ However, these differences were not accompanied by changes in histomorphometric parameters of bone resorption, nor did they persist after adjustment. Osteocytic RANKL expression in this cohort therefore appears to follow the circulating hormonal environment rather than marking a distinct resorptive phenotype.

Osteocytic RANKL expression was higher in biopsies showing marrow fibrosis, which was more frequent in OM-Al. Because fibrotic marrow contains RANKL-expressing stromal cells,^39^ the association between fibrosis and osteocytic RANKL may partly reflect the broader tissue microenvironment. However, three observations suggest that fibrosis alone does not account for the difference between groups: its extent was limited even in OM-Al (median Fb.V/TV, 0.11%), Fb.V/TV did not correlate with osteocytic RANKL expression within this group, and fibrosis was not independently associated with RANKL expression in the multivariable analysis. Marrow fibrosis is a well-recognized histological feature of sustained PTH excess, reflecting, at least in part, increased peritrabecular stromal cell proliferation.^42–44^ Its greater frequency in OM-Al is therefore consistent with the higher PTH levels observed in this group compared with OM+Al.

Taken together, these observations describe two phenotypes that differ less in the amount of unmineralized matrix than in the state of the cells embedded within it. OM-Al presents a hormonally driven, metabolically active osteoid in which osteocytes retain an extensive canalicular network and express the phosphate-handling and matrix-interaction proteins of that compartment. In OM+Al, a sparser canalicular network coexists with a selectively reduced osteocytic expression profile and a phosphate-rich milieu in which OPN accumulates, a combination compatible with an osteocyte constrained both structurally and functionally at the mineralizing surface. Which elements of this picture are caused by Al, which are mediated by the hormonal changes that Al itself induces, and which belong to OM irrespective of Al cannot be fully resolved in a cross-sectional cohort of this size; the multivariable models indicate where that boundary may lie rather than establishing it.

This study has several limitations. Its cross-sectional design precludes establishment of causal relationships between Al deposition and the observed osteocyte-related alterations. The relatively small sample size reflects the difficulty of obtaining adequate undecalcified bone biopsies in CKD patients. In addition, canalicular analyses were performed in a limited number of representative samples because of the technical complexity of the modified Ploton staining method. Furthermore, analyses were restricted to trabecular bone, since cortical thinning, increased porosity, trabecularization, and sectioning artifacts may impair reliable cortical compartment evaluation in CKD bone biopsies. Serum FGF23 was not measured. Although multivariable models were used to account for PTH, ALP and P the small sample size limits their statistical power, and no correction for multiple comparisons was applied, so the present findings should be regarded as exploratory and require confirmation in independent cohorts.

Two analyses that would have separated Al from its hormonal context more cleanly were not possible. Al involvement was essentially uniform among OM+Al biopsies, leaving no gradient of Al burden to correlate with the osteocyte findings, so a dose-response relationship remains to be examined in cohorts with a wider spectrum of deposition. A comparison restricted to PTH-matched cases of mixed ROD with and without Al was also unfeasible, because the database search that generated this cohort retrieved only biopsies with a histological diagnosis of OM. Finally, the healthy individuals were younger than the patients with OM and lacked serum biochemistry, so those comparisons should be read as descriptive.

Despite these limitations, this study provides novel evidence that CKD-associated OM exhibits distinct osteocyte-related and canalicular phenotypes according to Al deposition. Beyond impaired mineralization alone, Al-associated OM may involve structural and functional osteocyte abnormalities that contribute to altered matrix organization and skeletal dysfunction. These findings expand the understanding of CKD-associated OM and support a potential role for osteocyte dysfunction in the pathophysiology of Al-related bone disease.

### Conclusion

Aluminum deposition defines a distinct osteocytic phenotype in CKD-associated OM. While disease without metal accumulation displays a hormonally driven, metabolically active osteoid microenvironment, Al deposition selectively suppresses key osteocytic proteins and induces lacuno-canalicular disorganization. These findings demonstrate that the metal directly impairs local osteocyte architecture and function beyond causing a mechanical defect in mineralization.

## Supplementary Material

Supplementary_Material_R2_ziag167

## Data Availability

The data that support the findings of this study are available from the corresponding author upon reasonable request.
