# Supplementary material for: Osteocytic alterations in CKD-associated osteomalacia appear to differ according to aluminum deposition, suggesting distinct microenvironmental and canalicular phenotypes
Source: JBMR Plus. 2026 Oct 9;10(11):ziag167. doi: 10.1093/jbmrpl/ziag167 (PMC13695956; doi:10.1093/jbmrpl/ziag167)
Supplement: Supplementary file 1 [file supplementary_material_r2_ziag167.docx]

## **Supplementary Material**

## Supplementary Table S1. Multiple linear regression: aluminum-group effect after adjusting for PTH, phosphorus, and alkaline phosphatase

| **Protein** | **n** | **β (group)** | **95% CI** | **p (group)** | **R²** |
| --- | --- | --- | --- | --- | --- |
| **CD44 (global)** | **24** | **+18.64** | **8.03 to 29.24** | **0.0016** | **0.74** |
| RANKL (global) | 24 | +8.50 | −1.82 to 18.81 | 0.10 (ns) | 0.20 |
| OPN (global) | 21 | −9.38 | −23.36 to 4.61 | 0.17 (ns) | 0.30 |
| **CD44 (mineralized)** | **21** | **+33.01** | **12.08 to 53.94** | **0.0041** | **0.63** |
| CD44 (osteoid) | 21 | +4.73 | −18.16 to 27.62 | 0.67 (ns) | 0.50 |
| **FGF23 (osteoid)** | **21** | **+17.17** | **3.49 to 30.85** | **0.0171** | **0.50** |
| **MEPE (osteoid)** | **21** | **+20.40** | **0.33 to 40.48** | **0.0468** | **0.39** |
| E11 (osteoid) | 21 | +27.05 | −6.12 to 60.23 | 0.10 (ns) | 0.21 |
| RANKL (global), fibrosis added | 24 | +8.41 | −2.19 to 19.01 | 0.1127 (ns) | 0.21 |

Supplementary Table S1. Multiple linear regression: aluminum-group effect after adjusting for PTH, phosphorus, and alkaline phosphatase. β = adjusted effect of aluminum group (OM-Al vs. OM+Al), reference = OM+Al. All variance inflation factors < 2 (no meaningful multicollinearity). CD44-global bootstrap (2000 resamples) significant in 87% of resamples. Models run in GraphPad Prism (v10). Abbreviation: ns, not significant. The last row adds fibrosis volume (Fb.V/TV) to the model for global RANKL; in that model Fb.V/TV itself was not significant (β=+0.80; p=0.6884).

## Supplementary Table S2. Osteocyte protein expression in healthy individuals, OM-Al and OM+Al — parameters with a significant Kruskal–Wallis test

| **Protein** | **Healthy individuals, median (n=33)** | **OM-Al (n=15)** | **OM+Al**  **(n=12)** | **Kruskal–Wallis p** |
| --- | --- | --- | --- | --- |
| RANKL (%) | 4.07 (32) | 4.40 | 2.35 | 0.0326 |
| CD44 (%) | 14.90 (29) | 37.22 | 15.63 | <0.0001 |
| OPG (%) | 1.88 (31) | 8.50 | 5.00 | 0.0021 |
| E11 (%) | 0.14 (21) | 9.70 | 9.20 | <0.0001 |
| PHEX (%) | 3.76 (29) | 7.85 | 11.00 | 0.0033 |
| OPN (%) | 25.75 (20) | 40.90 | 57.40 | <0.0001 |
| Apoptosis (%) | 0.00 (33) | 0.80 | 0.41 | 0.0051 |

Values are medians. This table summarizes the seven parameters shown in Figure 3 that differed significantly (p<0.05) between healthy individuals, OM-Al and OM+Al. The number of healthy individuals contributing to each reaction is given in parentheses and varies according to the material available for each antibody. See Discussion for interpretation.

## Figure S1. Global osteocyte protein expression — individual data points (replaces/supplements Table 3)

## Figure S2. Osteocyte protein expression in mineralized bone — individual data points (replaces/supplements Table 4)

## Figure S3. Osteocyte protein expression in osteoid matrix — individual data points (replaces/supplements Table 5)

## Figure S4. Correlation between serum PTH and global osteocyte protein expression

Former Supplementary Figure S5 has been moved to the main manuscript as Figure 3. Former Supplementary Figure S6 showed a subset of the same panels and has not been duplicated; the corresponding values are given in Supplementary Table S2.
